# Co‐doping Strategy for Developing Perovskite Oxides as Highly Efficient Electrocatalysts for Oxygen Evolution Reaction

**DOI:** 10.1002/advs.201500187

**Published:** 2015-09-27

**Authors:** Xiaomin Xu, Chao Su, Wei Zhou, Yinlong Zhu, Yubo Chen, Zongping Shao

**Affiliations:** ^1^ State Key Laboratory of Materials‐Oriented Chemical EngineeringCollege of Chemistry and Chemical EngineeringNanjing Tech UniversityNo. 5 Xin Mofan RoadNanjing210009P. R. China; ^2^ Department of Chemical EngineeringCurtin UniversityPerthWestern Australia6845Australia; ^3^ State Key Laboratory of Materials‐Oriented Chemical EngineeringCollege of EnergyNanjing Tech UniversityNo. 5 Xin Mofan RoadNanjing210009P. R. China

**Keywords:** doping, electrocatalysis, oxygen evolution reaction, perovskite, water splitting

## Abstract

**A synergistic co‐doping strategy** is proposed to identify a series of BaCo_0.9–*x*_Fe*_x_*Sn_0.1_O_3–δ_ perovskites with tunable electrocatalytic activity for the oxygen evolution reaction (OER). Simply through tailoring the relative concentrations of less OER‐active tin and iron dopants, a cubic perovskite structure (BaCo_0.7_Fe_0.2_Sn_0.1_O_3–δ_) is stabilized, showing intrinsic OER activity >1 order of magnitude larger than IrO_2_ and a Tafel slope of 69 mV dec^−1^.

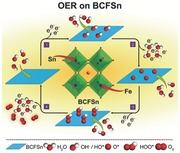

The design of cost‐effective and highly efficient catalysts for a wide range of electrochemical energy storage applications remains a key element in the societal pursuit of sustainable energy.^1^, ^2^, ^3^ In particular, the electrocatalytic splitting of water to generate hydrogen and oxygen enables the storage of a large amount of energy.^4^, ^5^, ^6^ However, the oxygen evolution reaction (OER) at the anode of a water electrolyzer is kinetically hampered by a complex four‐electron oxidation process and therefore requires a considerable overpotential (η) that could cause significant losses to the overall efficiency of water splitting. To afford fast kinetics and low overpotential in practical applications, noble metal oxide catalysts (e.g., IrO_2_ and RuO_2_) are often involved,^7^, ^8^ but the high cost and scarcity of precious metals hinder their large‐scale use. Furthermore, these precious‐metal catalysts suffer from poor durability over long‐term operations.^9^, ^10^, ^11^ Therefore, it is of prime importance to develop low‐cost and earth‐abundant alternatives with comparable or even better catalytic activity and improved stability than state‐of‐the‐art precious metal catalysts to achieve energy production on a large scale.

Noble‐metal‐free perovskite oxides, with a general formula of ABO_3_, where A is commonly a rare‐earth or alkaline‐earth metal and B a transition metal, possess great structural flexibility and thus have been extensively studied for a myriad of applications.^12^, ^13^, ^14^, ^15^ Most recently, their role as an OER electrocatalyst has gained renewed attention,^16^, ^17^, ^18^, ^19^, ^20^, ^21^, ^22^, ^23^, ^24^, ^25^ ever since the seminal research work of Bockris and Otagawa peroformed during the 1980s.^26^, ^27^ Noticeably, Ba_0.5_Sr_0.5_Co_0.8_Fe_0.2_O_3‐δ_ (BSCF), which was known as a high‐performance cathode material in intermediate‐temperature solid‐oxide fuel cells,^14^ has also been identified as a highly active OER catalyst operating at room temperature with much higher intrinsic activity than that of IrO_2_ as determined from a design model based on molecular orbital principles.^16^ Unfortunately, BSCF was later found to readily undergo surface amorphization after prolonged potential cycles under OER conditions.^28^, ^29^ Continued work is thus desirable to improve the design of perovskite oxides with enhanced catalytic activity and improved durability.

Doping of A‐ or B‐site cations in the perovskite structure has been reported to be an effective way to enhance OER catalysis.^30^, ^31^ Raabe et al. improved the OER electrocatalytic activity of PrMnO_3_ perovskites by doping Ca into the Pr site,^30^ and Tiwari et al. reported the enhancement of the OER activity of La_0.8_Sr_0.2_CoO_3_ with B‐site substitution of nickel or iron for cobalt.^31^ In this work, we report a series of BaCo_0.9‐*x*_Fe*_x_*Sn_0.1_O_3‐δ_ (BCFSn, *x* = 0.2, 0.3, 0.4, denoted, respectively, as BCFSn‐721, BCFSn‐631, BCFSn‐541) perovskite oxides through co‐doping BaCoO_3‐δ_ parent oxide with iron and tin for highly efficient OER electrocatalysis in alkaline media (4OH^−^ → O_2_ + 2H_2_O + 4e^−^). Ferrite perovskites were previously reported to be less OER‐active than cobaltite perovskites,^26^, ^27^ while tin‐containing perovskites showed no appreciable activity toward OER.^32^ By co‐doping these two metals with Sn at a minimal concentration, a cubic‐phase perovskite structure is stabilized with tunable OER activity, which is viable through tailoring the concentration of Fe dopant. This leads to the development of BaCo_0.7_Fe_0.2_Sn_0.1_O_3‐δ_ (BCFSn‐721) with superior OER electrocatalytic performance that is presumably attributable to the co‐doping effect.

BCFSn perovskite oxides with different Sn and Fe doping concentrations were synthesized by conventional high‐temperature solid‐state reactions and their phase structures were analyzed by X‐ray diffraction (XRD). The parent oxide BaCoO_3‐δ_ displayed a complicated lattice structure (Figure S1, Supporting Information), composed mainly of a hexagonal phase (space group: *P6_3_*/*mmc*) similar to 12HBaCoO_2.6_,^33^ with a small amount of an orthorhombic phase.^34^ Doping of the cobalt site in BaCoO_3‐δ_ with tin alone or iron alone failed to induce the formation of an oxygen vacancy‐disordered cubic phase (Figure S1, Supporting Information). Co‐doping these two metals, surprisingly, facilitated the formation of a cubic perovskite structure (space group: *Pm*–3*m*) as is evidenced by the typical XRD profiles shown in **Figure** **1**a. Such a cubic structure can be well maintained regardless of the variation in the iron dopant concentration when the structure is doped with an optimal amount of tin (Figure 1a). No extra characteristic peaks emerged from any of the three codoped perovskite oxides, suggesting that there were no impurities in these three products. Rietveld refinement reconfirmed the structure of each composition and reasonably good fitting was obtained based on a cubic symmetry (Figure S2 and Table S1, Supporting Information), which is consistent with previously reported results.^35^, ^36^ The above results revealed that co‐doping with Sn and Fe is indispensable for the stabilization of a cubic phase perovskite structure. The surface areas of the as‐prepared BCFSn powders were measured by nitrogen adsorption/desorption isotherms and calculated based on the Brunauer–Emmett–Teller (BET) method. All samples investigated in this study possessed similar surface areas of ≈1 m^2^ g^−1^ (Table S2, Supporting Information). In addition, these oxides were pore‐free, as evidenced by their broad Barrett–Joyner–Halenda (BJH) pore size distribution curves and low pore volumes as obtained from the desorption branch of the nitrogen sorption isotherms (Figures S3 and S4, Table S2, Supporting Information). Representative field‐emission scanning electron microscopy (FE‐SEM) images for BCFSn perovskites are shown in Figure S5, Supporting Information, at different magnifications. Particles of several micrometers were clearly observed and no obvious morphological distinction between these oxides was found. The oxygen nonstoichiometries of BCFSn oxides were determined to be ≈0.30 using iodometric titration (Table S3, Supporting Information). From the X‐ray photoelectron spectroscopy (XPS) survey shown in Figure S6, Supporting Information, the presence of Ba, Co, Fe, and Sn in the perovskites was demonstrated. High‐resolution XPS spectra revealed that the surface Co species were mainly in the oxidation state of approximately 3.0+ for all three BCFSn perovskite oxides (Figure 1b). It is noteworthy that the simultaneous deconvolution of both Co 2p and Ba 3d in (Ba, Co)‐containing perovskites was required due to the overlap between the Co 2p and Ba 3d peaks.^37^, ^38^, ^39^ The surface oxidation state of Sn in the three BCFSn perovskites is 4.0+, whereas that of Fe is difficult to determine from XPS but is estimated to be ≈4.0+ to maintain electrical neutrality (Figure S7, Supporting Information).^40^ Above results demonstrated that solid‐state reactions produced phase structures, surface areas, morphologies, oxygen nonstoichiometries, and surface Co oxidation states that are substantially invariant among the different BCFSn perovskites reported herein, thus enabling their systematic evaluation as electrocatalysts for oxygen evolution.

**Figure 1:**
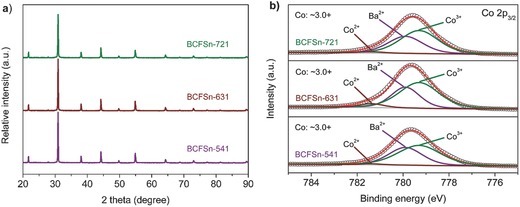
a) XRD patterns of BCFSn perovskites. b) High‐resolution XPS spectra of Co 2p_3/2_ core levels of BCFSn perovskites.

The catalytic activity of BCFSn perovskites for OER was examined in a 0.1 m KOH solution using a typical rotating disk electrode (RDE) technique.^39^ Cyclic voltammetry (CV) measurements reveal that BCFSn‐721 exhibits the lowest onset potential (≈1.53 V) with respect to a reversible hydrogen electrode (RHE) among the various BCFSn catalysts (**Figure** **2**a), indicating its high OER activity. This value is comparable to that of a high‐surface‐area commercial IrO_2_ catalyst (≈1.47 V vs RHE, Figure S8, Supporting Information), which serves as the benchmark for electrocatalysis in OER.^7^, ^16^ The OER activity of the glassy carbon (GC) substrate and conductive carbon (61.5 m^2^ g^−1^, Figure S9, Supporting Information) added to improve the conductivity of oxide electrodes was also measured, ensuring negligible background contributions from the GC disk and carbon. BCFSn‐541 and BCFSn‐631 both display comparable OER activity with somewhat larger onset potentials of ≈1.55 and ≈1.58 V vs RHE, respectively. Figure S10, Supporting Information, gives a more clear illustration of this observation, where OER activity was normalized by the catalyst loading and surface area of each perovskite oxide to gain the mass activity and specific activity, the latter known as intrinsic OER activity.^16^, ^41^ Based upon this, Tafel plots of BCFSn catalysts were plotted in Figure 2b together with IrO_2_, the activity of which is consistent with earlier reports.^7^, ^16^ As is clearly shown in Figure 2b, BCFSn catalysts were more active than IrO_2_ on a surface area basis, regardless of their variation in compositions, with intrinsic OER activity at least one order of magnitude higher than IrO_2_. Moreover, the Tafel slopes of the three BCFSn catalysts are 76, 68, and 69 mV dec^−1^ for BCFSn‐541, BCFSn‐631, BCFSn‐721, respectively (Table S4, Supporting Information). These values are in line with those of cobalt‐based perovskites as reported by Bockris and Otagawa.^26^ The analogous Tafel slopes of the three BCFSn catalysts (≈70 mV dec^−1^) compared to IrO_2_ (63 mV dec^−1^) indicate that BCFSn perovskites have outstanding OER kinetics and can therefore serve as highly efficient electrocatalysts that are competitive with some currently known high‐performance OER catalysts (Table S5, Supporting Information).^16^, ^17^, ^20^

**Figure 2:**
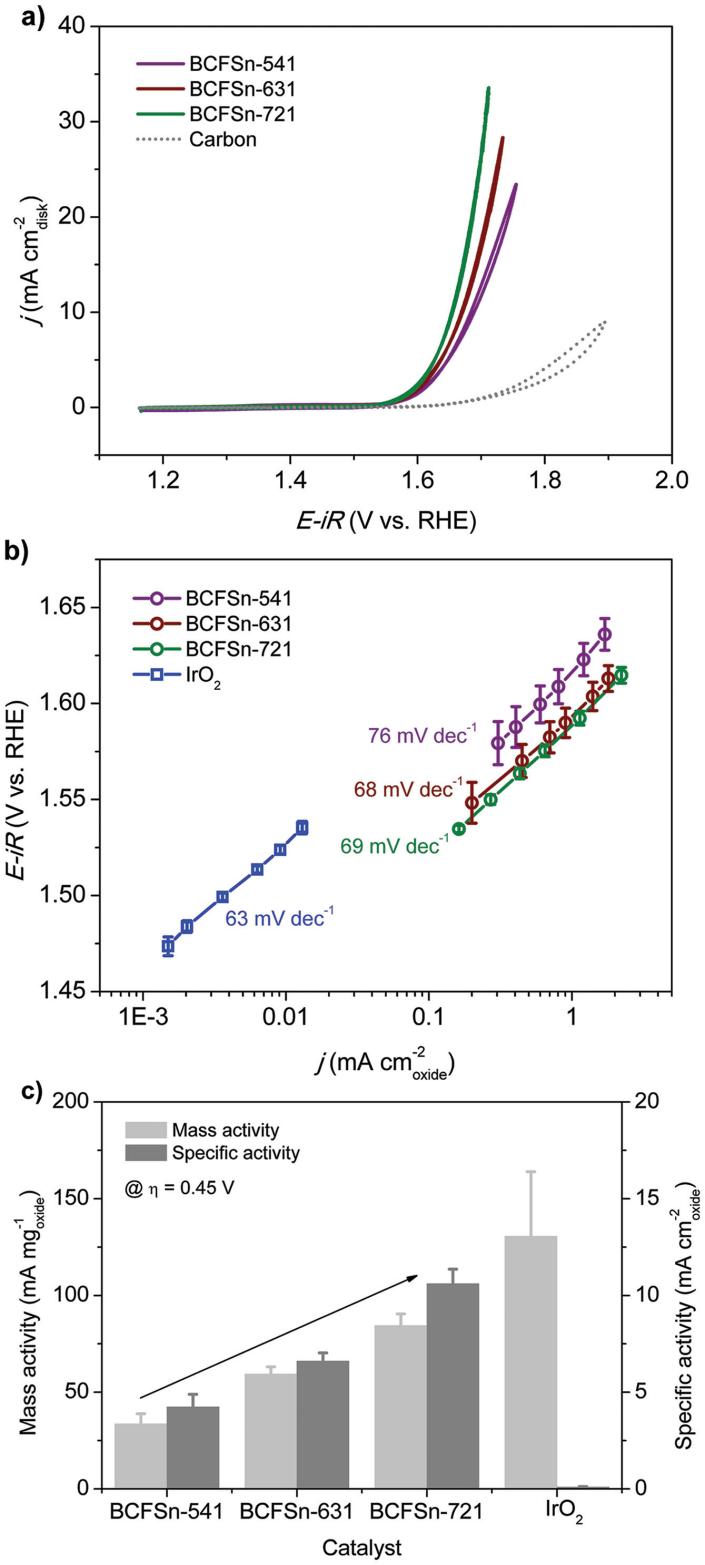
a) CV scans of BCFSn catalysts (catalyst loading of 0.232 mg_oxide_ cm^−2^_disk_), where measurements were performed on an RDE (1600 rpm) in 0.1 m KOH with a scanning rate of 10 mV s^−1^. The background OER activity of a carbon‐supported GC electrode is shown for reference. b) Tafel plots of BCFSn catalysts. c) Mass and specific activity of BCFSn catalysts at an overpotential of 0.45 V; the arrow indicates an ascending trend for the OER activity of BCFSn perovskites with decreasing iron dopants. The OER activity of commercial IrO_2_ (catalyst loading of 0.058 mg_oxide_ cm^−2^_disk_) is shown for comparison. Error bars represent standard deviations from at least three independent measurements.

The results shown in Figure 2a,b signify the high efficiency of BCFSn perovskites as OER electrocatalysts. We further report that their intrinsic OER activity can be tuned by tailoring the relative concentrations of the B‐site ions, namely, by increasing the concntration of the more OER‐active transition‐metal cation, e.g., Co cations in this case (or conversely, by decreasing the amount of the less OER‐active Fe dopant). This is despite the fact that the substitution of Co with Fe in (Pr_0.5_Ba_0.5_)CoO_3‐δ_ was reported to have a negligible effect on the OER activity.^17^ Evidence of this fact first came from the OER onset potential sequence of the three perovskites as discussed above. Further evaluation disclosed that the intrinsic OER activity of BCFSn perovskites with varied Co/Fe proportions follows the order of BCFSn‐541 < BCFSn‐631 < BCFSn‐721 (Figure 2b). This is particularly the case at more positive potential ranges where the anodic OER current increased rapidly. Specifically, a comparison of the mass and specific OER activity of various BCFSn electrocatalysts was made at η = 0.45 V (1.68 V vs RHE). As shown in Figure 2c, the OER mass activity for BCFSn‐541 was 33.7 mA mg^−1^_oxide_, followed by 59.4 and 84.5 mA mg^−1^_oxide_ for BCFSn‐631 and BCFSn‐721, respectively. Notably, with an increasing number of Co cations occupying the perovskite structure, the OER mass activity ascends by ≈1.8× and ≈2.5× for BCFSn‐631 and BCFSn‐721, respectively, with respect to BCFSn‐541. Likewise, the OER specific activity increased with a larger number of Co ions in BCFSn perovskites considering their similar surface areas. The OER specific activity was 4.2, 6.6, and 10.6 mA cm^−2^_oxide_ for BCFSn‐541, BCFSn‐631 and BCFSn‐721, respectively, again suggesting that BCFSn‐721 exhibited the highest OER activity. The minimal specific activity of the IrO_2_ catalyst was further elucidated at this selected overpotential. However, BCFSn catalysts underperform IrO_2_ with respect to mass activity as displayed in Figure 2c. Nonetheless, the mass activity of these BCFSn perovskites could be further enhanced to reach that of IrO_2_ by reducing their particle sizes via ball milling or developing hierarchical pore structures with larger surface areas.^16^, ^22^, ^23^

To gain insight into the origin of the quantitative trend in OER activity of BCFSn perovskites with altered co‐doping levels of iron and tin, we looked into the OER mechanism based upon the latest theoretical and experimental studies of Co‐based oxides for OER catalysis.^18^, ^19^, ^42^, ^43^ The electrocatalytic OER in alkaline electrolyte usually proceeds exclusively through four single‐electron charge‐transfer steps involving a sequence of reaction intermediates, HO*, O*, and HOO* (the asterisk indicates a bond to the catalyst surface), followed by the formation and desorption of the generated O_2_.^18^, ^19^, ^42^ For BCFSn perovskites with trivalent Co ions as the dominant Co species (Figure 1b), the adsorption of OH^−^ to form HO* involves the oxidation of Co^3+^ to Co^4+^, which would lead to inefficient adsorption, thereby making the formation of HO* rate‐limiting.^42^ Accordingly, we examined the capability of BCFSn perovskites to adsorb OH^−^ in alkaline solutions, as evidenced by Fourier transform infrared spectroscopy (FT‐IR) measurements in **Figure** **3**a. Broad IR bands centered at approximately 3420 cm^−1^, corresponding to H‐bonded stretching vibrations, appeared for all three BCFSn perovskites exposed to alkaline media.^44^ Markedly, BCFSn‐721 exhibited the strongest OH^−^ adsorption band, followed by BCFSn‐631 and BCFSn‐541, although as‐prepared BCFSn perovskites all tend to adsorb some water as revealed by thermogravimetry‐mass spectrometry (TG‐MS, Figure S11a, Supporting Information). Hence the rate of HO* formation follows the order of BCFSn‐541 < BCFSn‐631 < BCFSn‐721, and a similar trend is expected for the OER rate.

**Figure 3:**
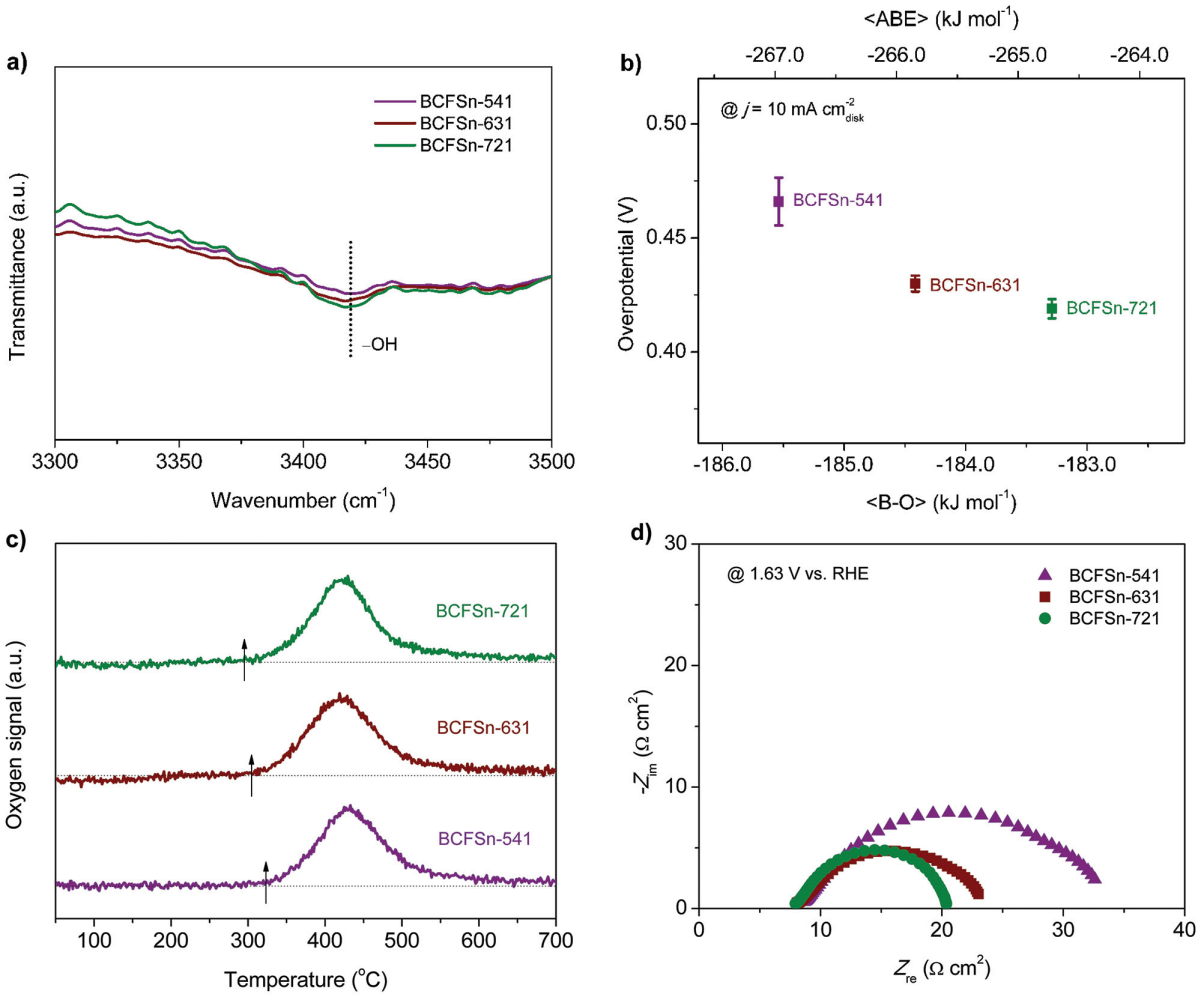
a) FT‐IR spectra of BCFSn perovskites after exposure to 0.1 m KOH. b) Correlation between the OER activity of BCFSn catalysts and the average B–O bond energy (〈B–O〉) or the average metal (A, B)–O bond energy (〈ABE〉) within each perovskite lattice. c) O_2_‐TPD profiles of BCFSn perovskites; arrows indicate the temperatures at which O_2_ desorption occurs. d) Electrochemical impedance spectra of BCFSn catalysts recorded at 1.63 V vs RHE under the influence of an AC voltage of 5 mV.

Recent research work has noted that lattice oxygen may participate in the formation of the O–O bond in the HOO* intermediate to play a partial role in the OER.^18^, ^19^ Based upon that idea, attempts were made to study the overall catalytic effect of B‐site cations (and A‐site cations, too) using the average B–O bond energy (〈B–O〉) or the average metal (A, B)–O bond energy (〈ABE〉) within the perovskite lattice,^45^ as estimated from the thermodynamic data.^46^ Figure 3b demonstrates the correlation between 〈B–O〉, or 〈ABE〉, and the OER activity of BCFSn perovskites, the latter being defined by the overpotential needed to oxidize water at a current density of 10 mA cm^−2^_disk_, a convention widely used in the OER literature.^47^ The negative values obtained for 〈B–O〉 and 〈ABE〉 indicate the exothermic character of the bond formation. The lower bond energy for BCFSn‐721 explains its easier release of lattice oxygen to facilitate the formation of HOO*. The relatively inferior OER performance for BCFSn‐631 and BCFSn‐541, as indicated by the higher overpotentials required to achieve 10 mA cm^−2^_disk_, can then be correlated with their higher 〈B–O〉 or 〈ABE〉.

Oxygen desorption, the last step needed to complete the OER cycle, is also an important factor for effective OER catalysis. The O_2_ desorption ability of BCFSn perovskites was characterized by performing O_2_ temperature‐programmed desorption (O_2_‐TPD, Figure 3c) and TG‐MS (Figure S11b, Supporting Information). The O_2_ desorption peak of BCFSn‐721 occurred at the lowest temperature, and those of BCFSn‐631 and BCFSn‐541 at more elevated temperatures. As a result, BCFSn‐721 shows enhanced OER activity.^23^ Charge transfer is a crucial factor for performance as efficient OER catalysts. As revealed by electrochemical impedance spectra (EIS) measurements in Figure 3d, the diameter of the semicircle for BCFSn‐541 is much larger than those of BCFSn‐631 and BCFSn‐721, which indicates a much higher charge transfer resistance for BCFSn‐541.^48^ In contrast, reducing the iron dopant concentration in BCFSn perovskites accelerated the charge transfer of the reactants to the catalysts. The rate of OER therefore follows the order of BCFSn‐541 < BCFSn‐631 < BCFSn‐721. Also of note is the increasing low‐temperature electrical conductivity resulting from the reduced doping with Fe that could benefit the OER (Figure S12, Supporting Information).

Stronger OH^−^ adsorption and greater O_2_ desorption capabilities, lower average metal–oxygen bond energy, better charge transfer ability, together with higher electrical conductivity, have given rise to a highly active OER catalyst, BCFSn‐721, among the various BCFSn perovskites reported herein. When regarding the trivalent Co cations as the major OER activity contributor, the exceptionally high intrinsic OER activity of BCFSn‐721 could be partially understood by employing the e_g_ activity descriptor.^16^ The electronic configuration of Co cations in BCFSn‐721 can be assigned as t_2g_^5^e_g_^≈1.0^ with e_g_ filling approaching unity under the assumption that the surface Co state in BCFSn‐721 is in an intermediate spin (IS) state at room temperatures. This, however, needs more experimental support despite the fact that previous cobalt‐based perovskites were reported to give the IS state under low temperatures.^49^, ^50^ Additionally, ambiguities could arise when adopting the e_g_ descriptor,^17^, ^19^ thus necessitating a case‐by‐case understanding of the perovskite oxides in catalyzing OER. In this study, when the less OER‐active ions, Fe and Sn, are doped into the BaCoO_3‐δ_ parent oxide, certain synergistic effect of these two elements is believed to be at work, as is the case of Mn and Cu in LaMn_1‐*x*_Cu*_x_*O_4_ for enhanced CO oxidation catalysis.^51^] **Figure** **4** shows the enhancement in OER activity of BCFSn‐721 compared to the parent oxide BaCoO_3‐δ_, and the Fe or Sn single‐doped BaCo_0.7_Fe_0.3_O_3‐δ_ (BCF) and BaCo_0.7_Sn_0.3_O_3‐δ_ (BCSn) oxides (Figures S1 and S4, Supporting Information). Doping Fe alone into the BaCoO_3‐δ_ oxide decreases the onset potential of OER to some degree with a concomitant decrease in the Tafel slope and increase in the intrinsic activity, whereas doping Sn alone merely shifts the onset potential with a larger Tafel slope and smaller intrinsic activity (Figure 4a,b, Figure S13, Supporting Information). By contrast, co‐doping with Fe and Sn to form a cubic‐phase perovskite structure improved the OER activity and kinetics significantly. Remarkably, the OER activity of BCFSn‐721 per Co atom is greater than that of BaCoO_3‐δ_ by ≈1 order of magnitude, while that of BCF or BCSn is larger only to a limited extent (Figure 4b). Additionally, at η = 0.45 V, both the mass and specific activity of BCFSn‐721 surpasses the sum of that of the un‐doped and single‐doped oxides (Figure 4c and Table S3, Supporting Information). The synergistic effect brought about by such a co‐doping strategy may arise from the stabilized oxygen vacancy‐disordered cubic phase structure of BCFSn‐721 while the iron dopant further modifies the surface properties. This hypothesis is further supported by the observation that the excessively tin doped BaCo_0.7_Fe_0.1_Sn_0.2_O_3‐δ_ (BCFSn‐712) with a secondary BaSnO_3_ phase showed inferior OER performance (Figures S1 and S14, Supporting Information). In addition, the cubic‐phase BCFSn‐541 and BCFSn‐631 perovskites, with fewer cobalt ions within their structure, exhibited OER activity on par or better than BaCoO_3‐δ_, BCSn and BCF (Figure S15 and Table S3, Supporting Information).

**Figure 4:**
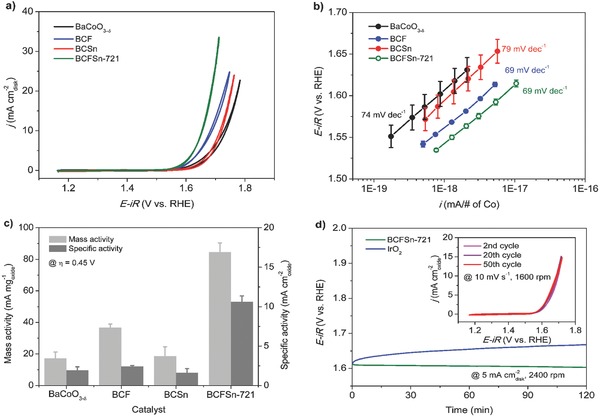
a) CV scans, b) OER activity normalized to the total Co atoms, and c) mass and specific activity at an overpotential of 0.45 V of BaCoO_3‐δ_, BCF, BCSn and BCFSn‐721 catalysts (catalyst loading of 0.232 mg_oxide_ cm^−2^_disk_) in 0.1 m KOH. d) Chronopotentiometry curves of BCFSn‐721 and commercial IrO_2_ catalysts (catalyst loading of 0.232 and 0.058 mg_oxide_ cm^−2^_disk_, respectively) at a constant current density of 5 mA cm^−2^_disk_ and 2400 rpm in 0.1 m KOH; the inset shows the 2nd, 20th, and 50th CV scans of BCFSn‐721 catalyst recorded at 10 mV s^−1^ and 1600 rpm.

Large‐scale application of water splitting requires the OER electrocatalysts to have high stability. When biased galvanostatically at a constant current density of 5 mA cm^−2^_disk_ on GC electrode, the BCFSn‐721 catalyst exhibited a nearly unchanged operating potential, at ≈1.61 V vs RHE in 0.1 m KOH, whereas IrO_2_ showed an apparent decline in OER activity as evidenced by the ascending overpotential needed to retain 5 mA cm^−2^_disk_ (Figure 4d). The inset in Figure 4d shows repeated CV scans of BCFSn‐721 and the 50th CV curve overlays almost exactly with the 2nd one. This confirms that BCFSn‐721 is highly stable to withstand operational conditions of the OER. BCFSn‐541 and BCFSn‐631 also exihited good stability during the same durability tests (Figures S16 and S17, Supporting Information).

In summary, solid‐state reactions were implemented in this work to fabricate a series of tin and iron codoped BCFSn perovskites with nearly identical phase purities, surface areas, morphologies and surface Co oxidation states. The OER activity of these perovskites could be tuned by simply modifying the concentrations of both dopants while stabilizing the cubic‐phase structure. Owing to their tunable OH^−^ adsorption and O_2_ desorption capability, average metal–oxygen bond energy, charge transfer ability, and electrical conductivity, a perovskite oxide with optimized doping of Fe and Sn, BCFSn‐721, was developed and showed intrinsic OER activity >1 order of magnitude larger than IrO_2_ in alkaline media and a small Tafel slope of 69 mV dec^−1^. Such remarkable OER activity may arise from a synergistic effect of the co‐doping of two less OER‐active elements, apart from an assumptive close‐to‐unity e_g_ filling of the OER‐active Co cations. Furthermore, BCFSn perovskites exhibited superior stability under OER conditions. Our approach provides a general route to the development of stabilized multication‐doped Co‐based perovskite catalyst structures, which holds promise for a variety of electrochemical energy storage applications.

## Supporting information

As a service to our authors and readers, this journal provides supporting information supplied by the authors. Such materials are peer reviewed and may be re‐organized for online delivery, but are not copy‐edited or typeset. Technical support issues arising from supporting information (other than missing files) should be addressed to the authors.

Supplementary
