# Supplementary material for: Co‐doping Strategy for Developing Perovskite Oxides as Highly Efficient Electrocatalysts for Oxygen Evolution Reaction
Source: Adv Sci (Weinh). 2015 Sep 27;3(2):1500187. doi: 10.1002/advs.201500187 (PMC5054898; doi:10.1002/advs.201500187)
Supplement: Supplementary file 1 — Supplementary [file ADVS-3-0m-s001.pdf]

Copyright WILEY-VCH Verlag GmbH & Co. KGaA, 69469 Weinheim, Germany, 2015.

ADVANCED SCIENCE
Open Access

Supporting Information

for *Adv. Sci.*, DOI: 10.1002/advs. 201500187

Co-doping Strategy for Developing Perovskite Oxides as Highly Efficient Electrocatalysts for Oxygen Evolution Reaction

*Xiaomin Xu, Chao Su, Wei Zhou, Yinlong Zhu, Yubo Chen, and Zongping Shao**

Copyright WILEY-VCH Verlag GmbH & Co. KGaA, 69469 Weinheim, Germany, 2013.

Supporting Information

Co-doping Strategy for Developing Perovskite Oxides as Highly Efficient Electrocatalysts for Oxygen Evolution Reaction

*Xiaomin Xu, Chao Su, Wei Zhou, Yinlong Zhu, Yubo Chen, Zongping Shao**

X. M. Xu, Prof. W. Zhou, Y. L. Zhu, Y. B. Chen
State Key Laboratory of Materials-Oriented Chemical Engineering, College of Chemistry & Chemical Engineering, Nanjing Tech University, No.5 Xin Mofan Road, Nanjing 210009, P.R. China
Dr. C. Su, Prof. Z. P. Shao
Department of Chemical Engineering, Curtin University, Perth, Western Australia 6845, Australia
Prof. Z. P. Shao
State Key Laboratory of Materials-Oriented Chemical Engineering, College of Energy, Nanjing Tech University, No.5 Xin Mofan Road, Nanjing 210009, P.R. China
E-mail: shaozp@njtech.edu.cn

I. Experimental Details

Catalyst synthesis. $BaCo_{0.9-x}Fe_xSn_{0.1}O_{3-\delta}$ (BCFSn, x = 0.2, 0.3, 0.4, denoted, respectively, as BCFSn-721, BCFSn-631, BCFSn-541), $BaCoO_{3-\delta}$, $BaCo_{0.7}Fe_{0.3}O_{3-\delta}$ (BCF), $BaCo_{0.7}Sn_{0.3}O_{3-\delta}$ (BCSn), and $BaCo_{0.7}Fe_{0.1}Sn_{0.2}O_{3-\delta}$ (BCFSn-712) oxides were fabricated using a solid-state reaction strategy reported previously.[1,2] Briefly, stoichiometric amounts of $BaCO_3$, Co_3O_4, Fe_2O_3, SnO_2 (all of analytical grade) were subjected to high energy ball milling (Fritsch Pulverisette 6) in acetone at 400 rpm for 40 min. The homogeneously dispersed mixture was then dried and calcined under various conditions. $BaCoO_{3-\delta}$ parent oxide and iron single-doped BCF oxide were calcined at 1000 °C for 10 h in air, while tin doped BCSn, BCFSn and BCFSn-712 oxides were calcined at 950 °C for 5 h in air, followed by further calcination at 1100 °C for 10 h in air. All the powders after heat treatment were finely ground using mortar and pestle prior to further characterization. Commercial IrO_2 (Aladdin Industrial Corporation) was purchased and used as received.

Basic Characterization. The crystal structures of as-prepared powders were investigated by room temperature X-ray diffraction (XRD, Rigaku Smartlab 3kW) with filtered Cu Kα radiation (λ = 1.5406 Å) operating at a tube voltage of 40 kV and current of 40 mA. Diffraction patterns were collected by step scanning in the 2θ range of 20–90° with intervals of 0.02°. More detailed structural information was obtained by Rietveld refinement of the XRD patterns with the GSAS-EXPGUI package. The surface area and pore size distribution were characterized by nitrogen adsorption-desorption tests (BELSORP II) using the Brunauer-Emmett-Teller (BET) and Barrett-Joyner-Halenda (BJH) methods, respectively. Approximately 2.0 g samples were weighed and degassed at 200 °C for at least 3 h before N_2 physisorption measurements at the temperature of liquid nitrogen (77 K). Particulate morphologies of the catalyst powders were examined by a field emission scanning electron microscope (FE-SEM, Hitachi S-4800). The room-temperature oxygen non-stoichiometry was measured by the iodometric titration method. The elemental composition and oxidation states

of the B-site cations of BCFSn perovskites were determined on an X-ray photoelectron spectrometer (XPS, PHI5000 VersaProbe) equipped with an Al Kα X-ray source. The binding energies were calibrated to the adventitious C 1s peak at 284.8 eV and data were fitted by the public software package XPSPEAK. The ability of BCFSn perovskites to adsorb OH^- after exposure to alkaline solutions was estimated from their Fourier transform infrared spectra (FT-IR, Nicolet iS10). The oxygen desorption capability of BCFSn perovskites was characterized using O_2-TPD measurements, which were performed up to 930 °C with a heating rate of 10 °C min^{-1} in pure argon (15 mL min^{-1}). The effluent gases were analyzed by a mass spectrometer (Hiden, QIC-20) to monitor the oxygen concentration. Thermogravimetric (TG) analysis was performed on a thermobalance (STA 449 *F3* Jupiter®, NETZSCH), and the qualitative determination of gas components emitted (H_2O and O_2) was conducted in a mass spectrometer (QMS 403D Aëolos®, NETZSCH). Approximately 10 mg of each BCFSn sample was heated from 50 °C to 930 °C with a heating rate of 10 °C min^{-1} in a flow of N_2 (20 mL min^{-1}). The electrical conductivity of BCFSn powders was measured in air atmosphere based on a four-probe DC configuration using a Keithley 2420 source meter within the temperature range of 300 °C to around room temperature.

Electrochemical measurements. Electrodes were prepared by a controlled drop-casting approach involving a glassy carbon (GC) electrode (0.196 cm^2, Pine Research Instrumentation), as described elsewhere.[3] The GC substrate was pre-polished to a mirror finish with 50 nm α-Al_2O_3 slurries on a polishing cloth and rinsed with deionized water prior to electrode preparation. For the preparation of catalyst inks, 10 mg of perovskite oxide powder, 10 mg of conductive carbon (Super P Li, Figure S9) and 100 μL of 5 wt% Nafion solution were dispersed in 1 mL of absolute ethanol by mild sonication to form a homogeneous ink. Then, a 5-μL aliquot of the as-prepared catalyst ink was transferred onto the surface of the GC substrate, yielding an approximate catalyst loading of 0.232 mg_{oxide} cm^{-2}_{disk}. Electrodes containing commercial IrO_2 were also prepared. Specifically, 2.5 mg of

IrO_2, 2.5 mg of conductive carbon and 100 μL of 5 wt% Nafion solution were dispersed in 1 mL of absolute ethanol by mild sonication to form a homogeneous ink. A 5-μL aliquot of the as-prepared catalyst ink was then transferred onto the surface of the GC substrate, yielding an approximate catalyst loading of 0.058 mg_{oxide} cm^{-2}_{disk}.

OER measurements were conducted at room temperature in a standard three-electrode electrochemical cell (Pine Research Instrumentation) in an RDE configuration with a CHI 760E bipotentiostat. All tests were performed in 0.1 M potassium hydroxide (KOH) electrolyte using a catalyst-modified GC working electrode with a platinum foil counter electrode and Ag/AgCl (4 M KCl) reference electrode. The system was purged with ultra-pure oxygen (99.999% purity) before each measurement for 30 min to ensure the O_2/H_2O equilibrium at 1.23 V vs. reversible hydrogen electrode (RHE).

Prior to OER characterization, the catalyst was electrochemically activated by running cyclic voltammetry (CV) at least five times until reproducible curves were observed. The activity of the catalyst for OER was measured by running CV at 10 mV s^{-1}. Electrochemical impedance spectra were recorded on the CHI 760E bipotentiostat with frequencies ranging from 100 kHz to 0.1 Hz at 1.63 V vs. RHE under the influence of an AC voltage of 5 mV. Throughout these measurements, the RDE was rotated at 1600 rpm to remove O_2 gas bubbles generated at the catalyst surface. The chronopotentiometry tests were conducted at a constant current density of 5 mA cm^{-2}_{disk} for 2 hours, where a higher rotation speed of 2400 rpm was adopted to guarantee sufficient removal of oxygen generated.

The OER kinetic current was capacitance-corrected by calculating the average of the anodic and cathodic scans of the CV data and then corrected for ohmic losses by subtracting the ohmic voltage drop from the measured potential using an electrolyte resistance determined by high-frequency AC impedance, where iR-corrected potentials are denoted as $E-iR$ (i as the current and R as the uncompensated ohmic electrolyte resistance, ~45 Ω). The mass activity and specific activity of OER were obtained from normalizing the kinetic current by the

catalyst loading and the surface area of each oxide calculated by the BET method. The activity of several oxide catalysts was also normalized to the total number of cobalt atoms present on the electrodes, which was calculated based on the catalyst mass loading and stoichiometry. Error bars represent standard deviations from at least three independent measurements.

A Ag/AgCl (4 M KCl) electrode was used as the reference electrode during the recording of electrochemical measurements, which was calibrated with respect to RHE (Figure S18). The calibration was performed in a high purity hydrogen saturated electrolyte with a platinum rotating disk electrode (0.126 cm^2, Pine Research Instrumentation) as the working electrode. CV was cycled at a scan rate of 1 mV s^{-1}, and the average of the two potentials at which the current crossed zero, which was approximately 0.962 V, was taken to be the thermodynamic potential for the hydrogen electrode reaction. The overpotential (η) was calculated according to the following formula: η (V) = E(RHE) − 1.23 V.

II. Supplementary Results

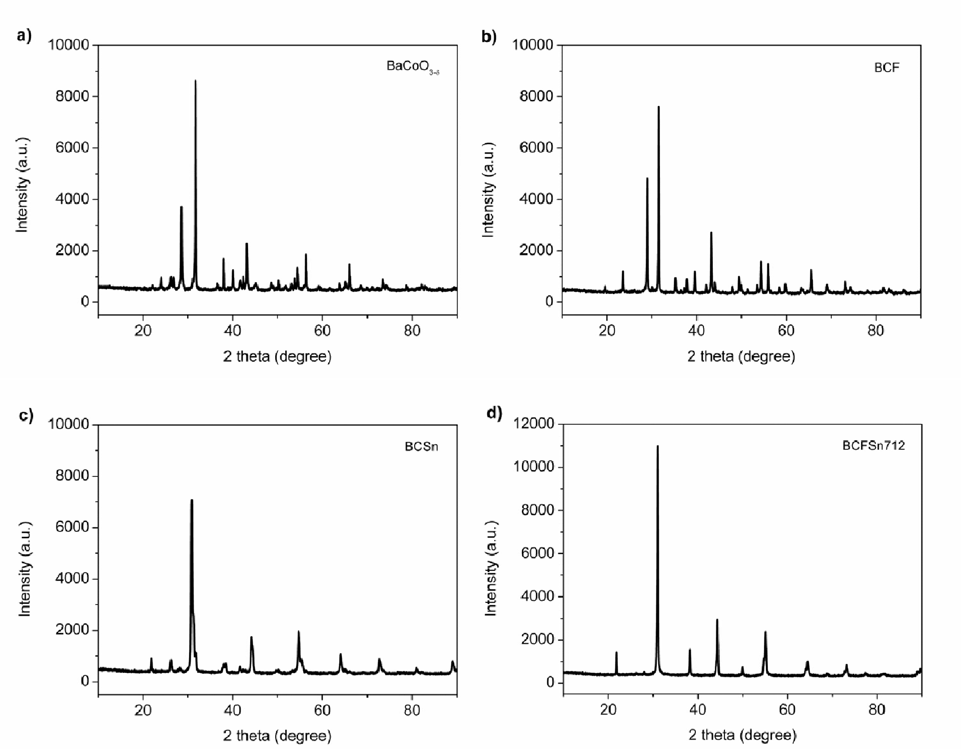

Figure S1. XRD patterns of a) $BaCoO_{3-\delta}$, b) $BaCo_{0.7}Fe_{0.3}O_{3-\delta}$ (BCF), c) $BaCo_{0.7}Sn_{0.3}O_{3-\delta}$ (BCSn) and d) $BaCo_{0.7}Fe_{0.1}Sn_{0.2}O_{3-\delta}$ (BCFSn-712).

XRD patterns reveal that the parent oxide $BaCoO_{3-\delta}$ (Figure S1a) displayed a complicated lattice structure consisting mainly of a hexagonal phase (space group: $P6_3/mmc$) similar to $12HBaCoO_{2.6}$,[4] with a small amount of an orthorhombic phase.[5] As shown in Figure S1b and S1c, doping iron or tin alone into the cobalt site of $BaCoO_{3-\delta}$ failed to promote the formation of oxygen vacancy-disordered cubic phase. However, co-doping these two metals (iron and tin) with optimized amount of tin successfully stabilized the cubic perovskite structure of $BaCoO_{3-\delta}$ as presented in Figure 1 in the main text, and this cubic structure can be well maintained with altered iron dopant while the tin dopant remained unchanged. In the case of excessively tin dopant (BCFSn-712), exsolution of a secondary $BaSnO_3$ phase occurred (Figure S1d), which is believed to show negligible OER activity.

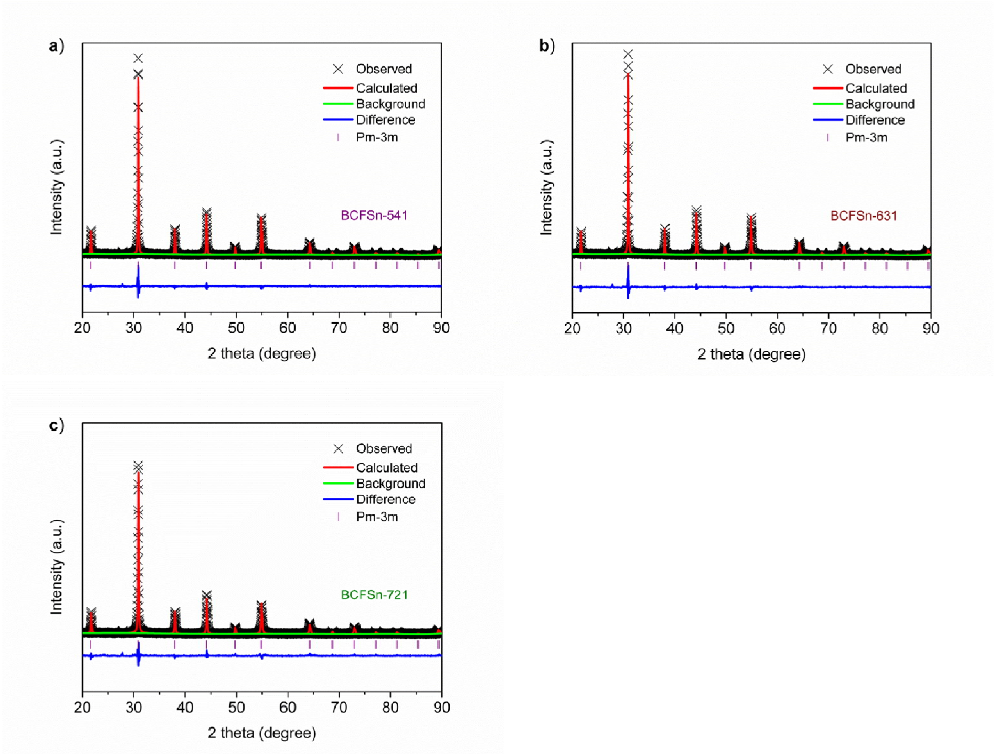

Figure S2. Rietveld refinement XRD patterns of a) BCFSn-541, b) BCFSn-631 and c) BCFSn-721.

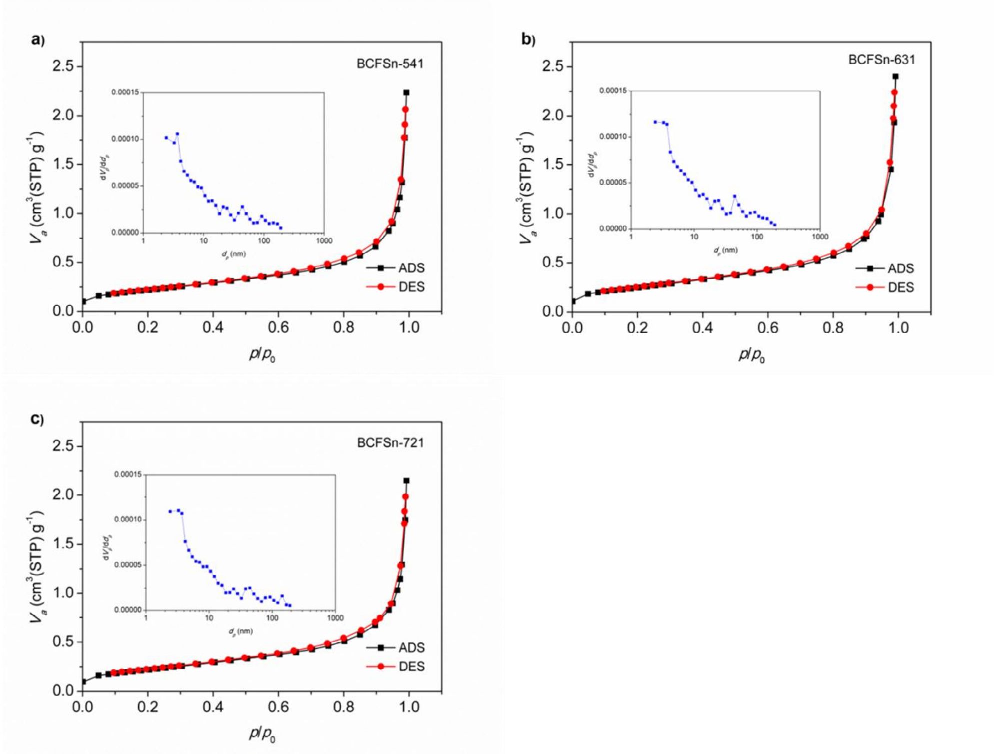

Figure S3. Nitrogen adsorption-desorption isotherms of as-prepared BCFSn powders, a) BCFSn-541, b) BCFSn-631 and c) BCFSn-721; the insets show the corresponding BJH pore size distribution curves obtained from the desorption branch of the nitrogen adsorption/desorption isotherms.

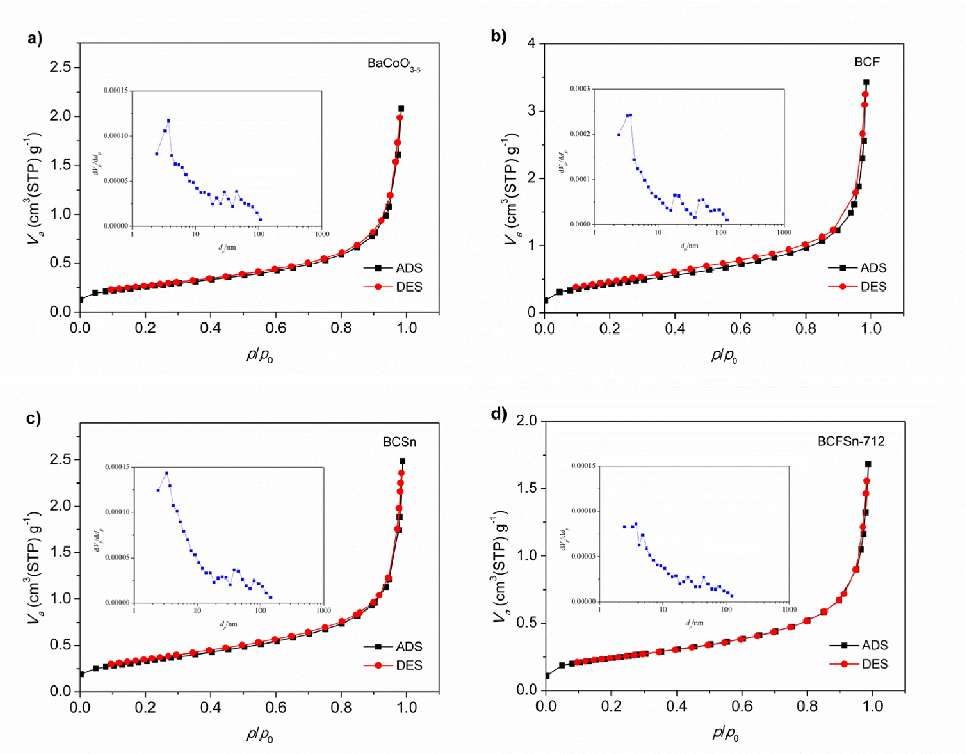

Figure S4. Nitrogen adsorption-desorption isotherms of a) $BaCoO_{3-\delta}$, b) BCF, c) BCSn and d) BCFSn-712; the insets show the corresponding BJH pore size distribution curves obtained from the desorption branch of the nitrogen adsorption/desorption isotherms.

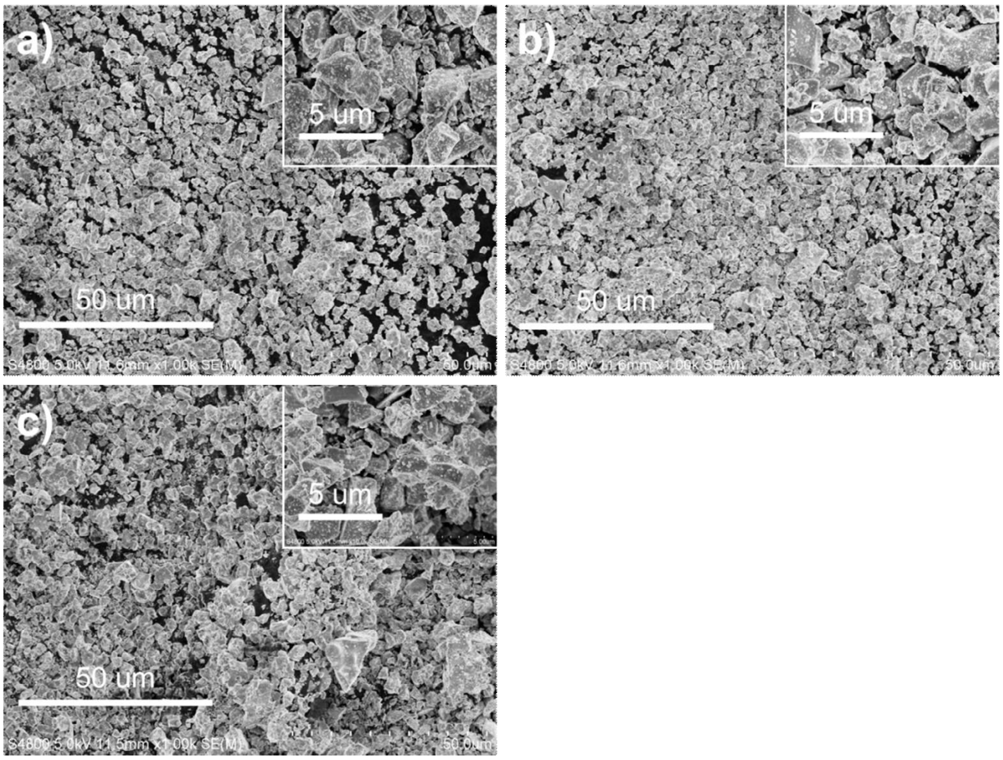

Figure S5. FE-SEM images of as-prepared BCFSn powders, a) BCFSn-541, b) BCFSn-631 and c) BCFSn-721; the insets show the corresponding magnified images.

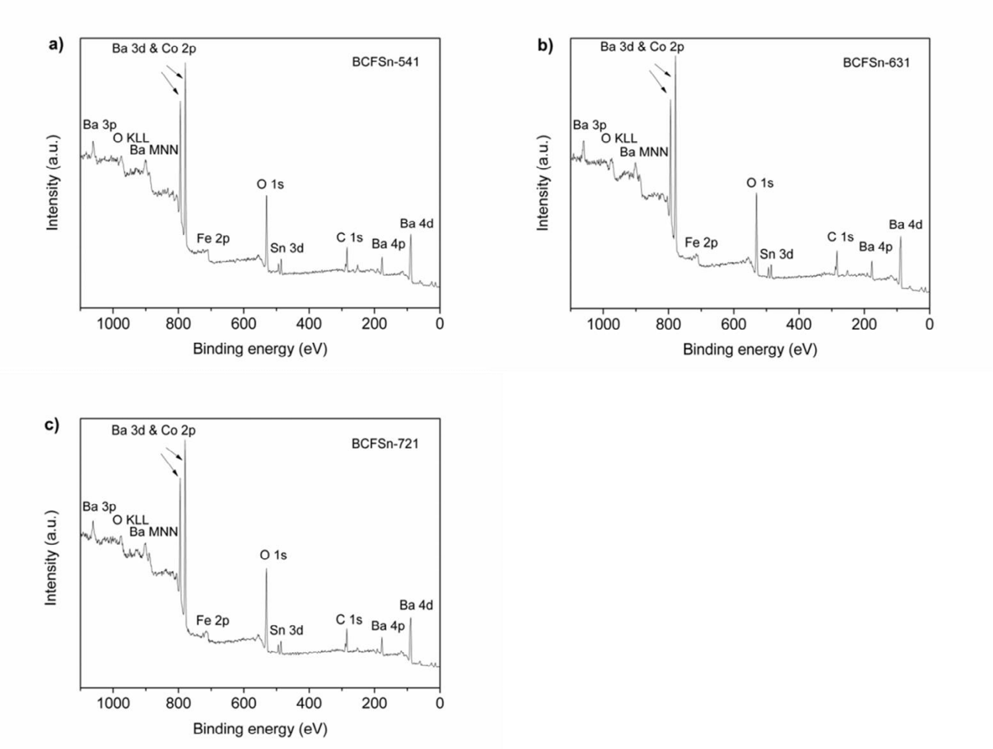

Figure S6. XPS survey spectra of as-prepared BCFSn powders, a) BCFSn-541, b) BCFSn-631 and c) BCFSn-721.

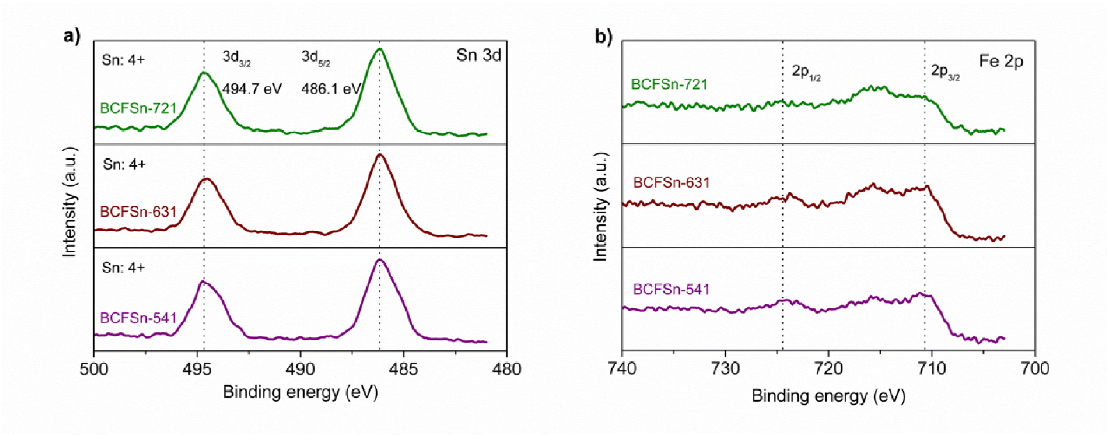

Figure S7. High-resolution XPS spectra of a) Sn 3d and b) Fe 2p core levels of BCFSn perovskites.

For various BCFSn perovskites, while the effect of Sn^{4+} doping on the spin state transition of cobalt and iron remains unknown, Sn^{4+} doping has proved to alter the average oxidation state of cobalt and iron, which turned out to be approximately 3.3 (see Table S3).[1,2] Although it is difficult to distinguish Fe^{3+} and Fe^{4+} from the Fe 2p XPS signal,[6] considering the ease of Fe ions to form B^{4+} (B cation in the ABO_3 structure) compared to Co,[7] it is reasonable to speculate that Fe ions are in the mixed oxidation states of 3+ and 4+ (mainly in 4+) and that Co ions are mainly in the oxidation state of ~3+. The latter hypothesis can be further supported by findings in other perovskites with similar compositions. For example, X-ray absorption near-edge structure (XANES) and XPS studies elucidated that the Co oxidation state in BSCF was partially reduced below 3+ and that the Fe oxidation state was partially oxidized above 3+.[8,9] Therefore, the Co $2p_{3/2}$ core-level spectra for BCFSn perovskites were deconvoluted based on Co^{2+} and Co^{3+} (Figure 1b in the main text).

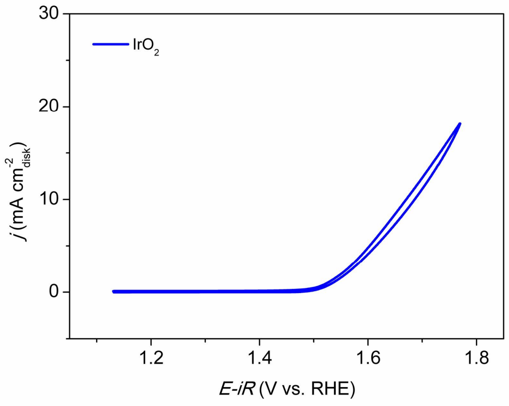

Figure S8. CV scan of commercial IrO_2 oxide (catalyst loading: 0.058 mg_{oxide} cm^{-2}_{disk}), where measurement was performed on an RDE (1600 rpm) in 0.1 M KOH with a scanning rate of 10 mV s^{-1}.

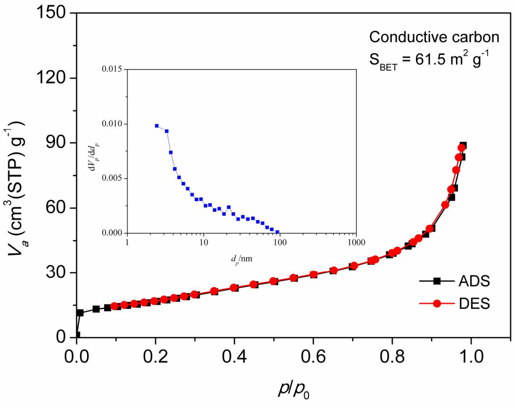

Figure S9. Nitrogen adsorption-desorption isotherms of conductive carbon (Super P Li); the inset shows the corresponding BJH pore size distribution curves obtained from the desorption branch of the nitrogen adsorption/desorption isotherms.

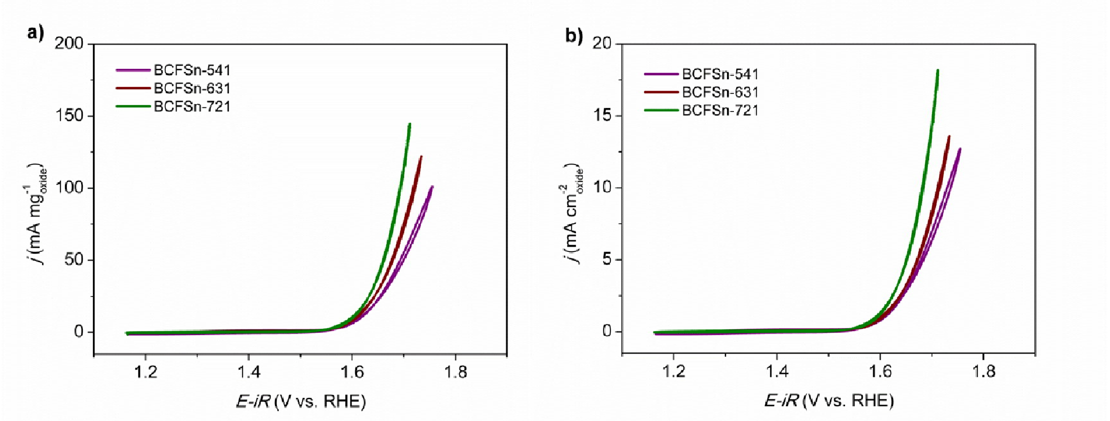

Figure S10. a) Mass activity b) specific activity of BCFSn and IrO_2 catalysts normalized, respectively, by the catalyst loading and the surface area of each catalyst.

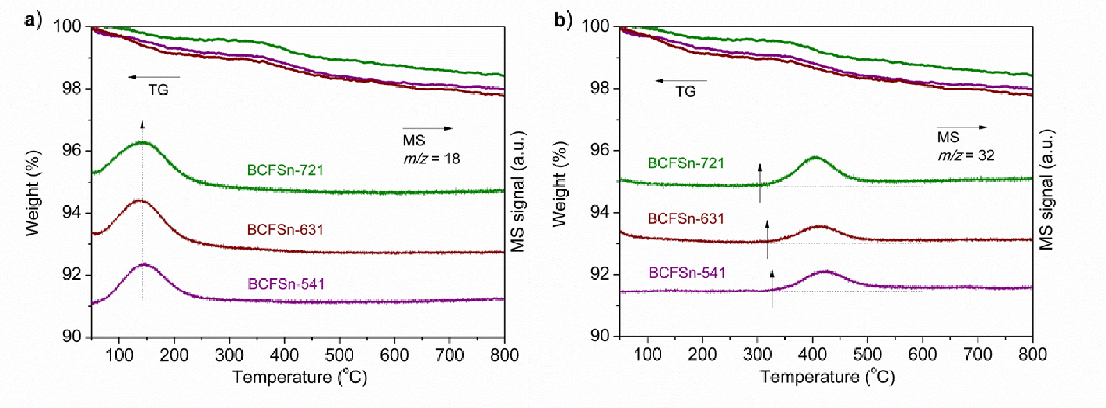

Figure S11. TG and MS analysis of BCFSn perovskites. a) m/z = 18 for the H_2O MS signal and b) m/z = 32 for the O_2 MS signal.

The dash arrow in Figure S11a indicates the detection of H_2O adsorbed at the same temperature for all three BCFSn perovskites, which suggests that as-prepared BCFSn perovskites are prone to adsorb some water. Such ability, however, does not interfere much with their ability to adsorb OH^- in alkaline solutions as shown in Figure 3a in the main text.
The arrows in Figure S11b indicate the temperatures at which O_2 desorption occurs and the result is in good agreement with that in Figure 3c in the main text.

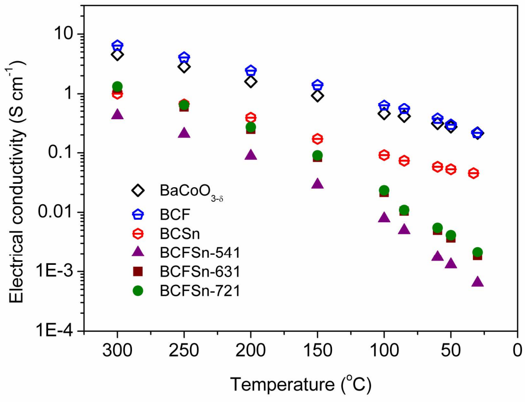

Figure S12. The electrical conductivity of $BaCoO_{3-\delta}$, BCF, BCSn, and BCFSn perovskites measured in air atmosphere at low temperatures.

As can be seen from Figure S12, the low-temperature electrical conductivity of the materials before and after doping is rather low, which is why conductive carbon is often introduced to the perovskite catalysts. The low-temperature electrical conductivity of BCFSn shows an increasing trend with a reduced doping amount of Fe. Given that the OER performance of BCFSn perovskites is evaluated under identical conditions, it is reasonalbe to establish a correlation between the electrical conductivity of BCFSn and their OER activity.
Surprisingly, the parent oxide $BaCoO_{3-\delta}$, and the Fe or Sn single-doped BCF and BCSn oxides, exhibit room-temperature electrical conductivity that is several orders of magnitude larger than that of BCFSn-721, but with much lower OER activity, as is demonstrated in the main text. This discrepancy can partially be understood considering that conductive carbon is mixed with the perovskite oxide in all the cases to improve the conductivity of the catalysts. Therefore, it is believed that, apart from electrical conductivity, certain synergistic effect is at work after co-doping $BaCoO_{3-\delta}$ with tin and iron to deliver an enhanced OER activity.

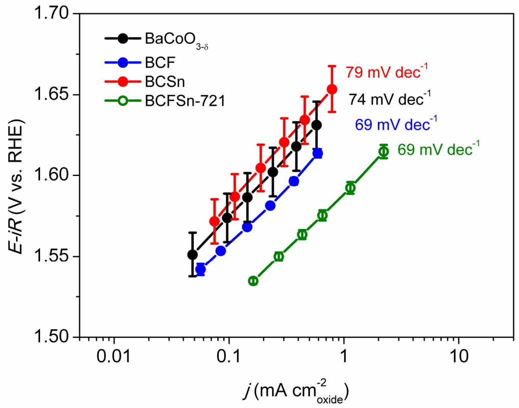

Figure S13. Intrinsic OER activity of $BaCoO_{3-\delta}$, BCF, BCSn and BCFSn-721 catalysts in 0.1 M KOH.

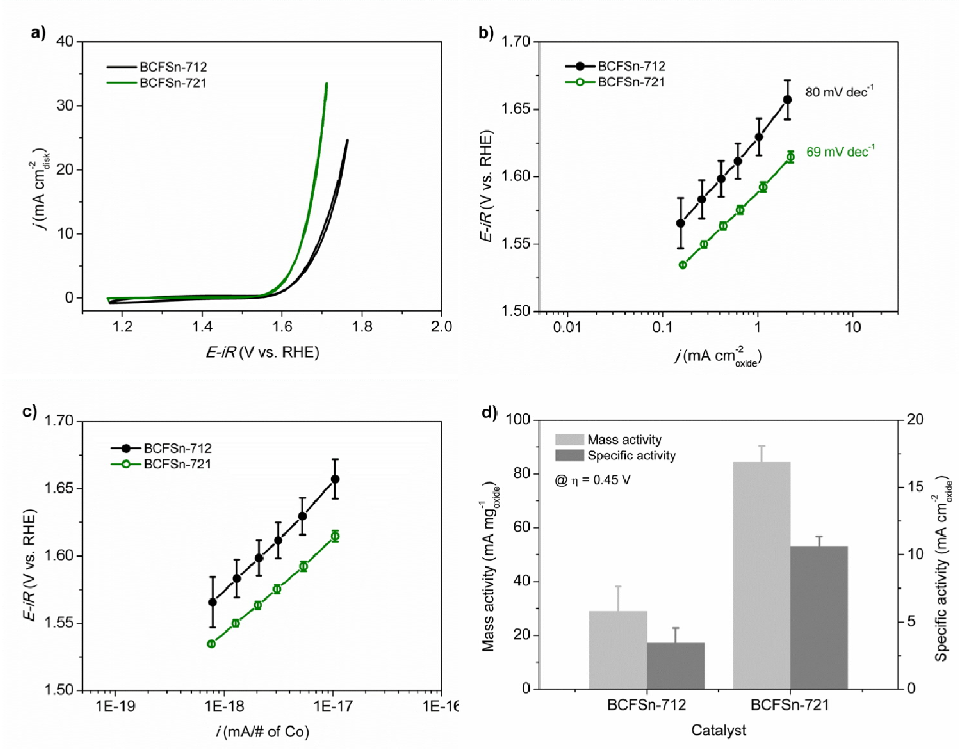

Figure S14. a) CV scans, OER activity normalized to b) the specific surface areas and c) the total Co atoms, and d) mass activity and specific activity at an overpotential of 0.45 V of BCFSn-712 and BCFSn-721 catalysts in 0.1 M KOH.

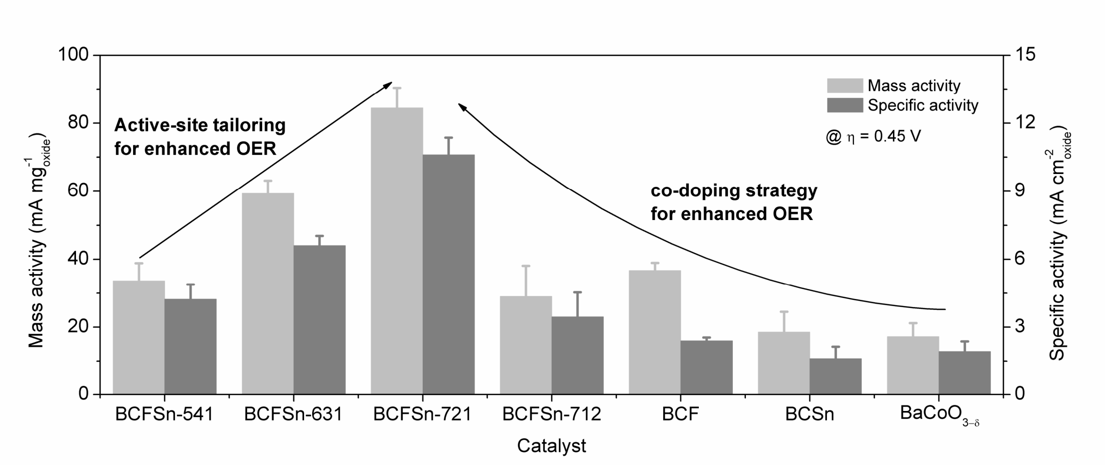

Figure S15. Mass and specific OER activity of BCFSn, $BaCoO_{3-δ}$, BCSn, BCF, BCFSn-712 catalysts at η = 0.45 V.

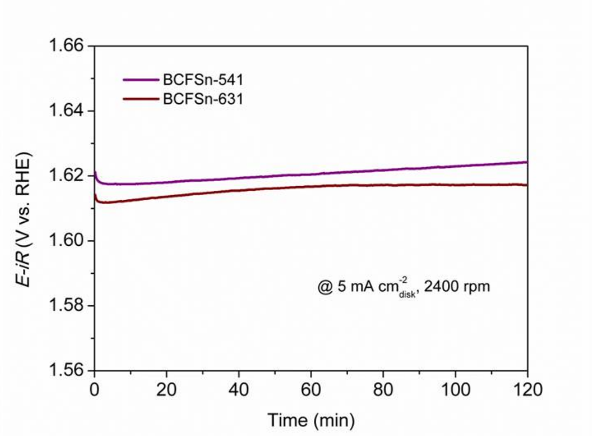

Figure S16. Chronopotentiometric responses of BCFSn-541 and BCFSn-631 catalysts at a constant current density of 5 $mAcm^{-2}{}_{disk}$ and 2400 rpm in 0.1 M KOH.

The potentials of the BCFSn electrocatalysts required to reach the current density of 5 mA $cm^{-2}{}_{disk}$ as shown in Figure S16 and Figure 4d in the main text, follow the order of BCFSn-721 < BCFSn-631 < BCFSn-541, also suggesting the OER activity sequence of BCFSn-541 < BCFSn-631 < BCFSn-721.

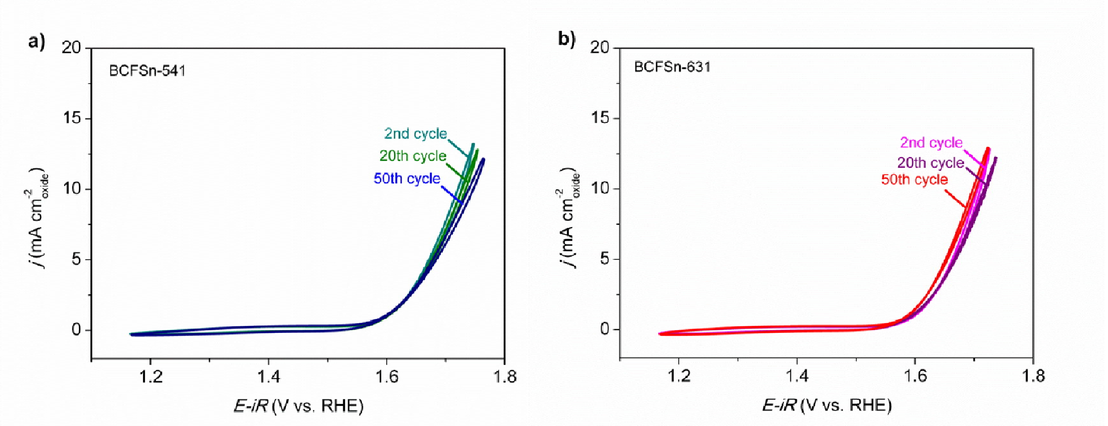

Figure S17. The 2nd, 20th and 50th CV scans of a) BCFSn-541 and b) BCFSn-631 catalysts recorded at 10 mV s^{-1} and 1600 rpm.

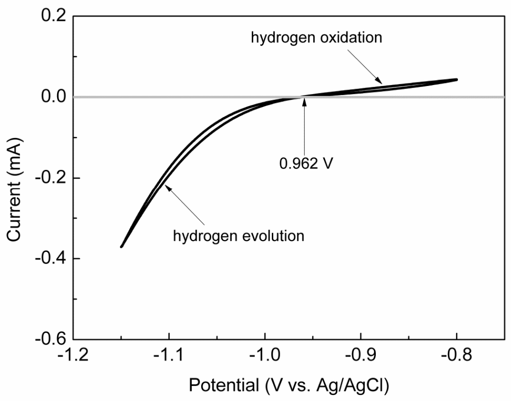

Figure S18. RHE calibration of the Ag/AgCl reference electrode in 0.1 M KOH.

Table S1. Rietveld refined lattice parameters and reliability factors for BCFSn perovskites.

Sample	Lattice parameter (Å)	χ^2	R_p (%)	R_{wp} (%)
BCFSn-541	4.099(1)	1.862	5.35	6.98
BCFSn-631	4.102(2)	1.860	5.18	6.72
BCFSn-721	4.102(9)	1.736	5.04	6.38

Table S2. Specific surface areas (A_s) and total pore volumes of as-prepared BCFSn, $BaCoO_{3-\delta}$, BCF, BCSn, BCFSn-712 powders and commercial IrO_2 measured by nitrogen adsorption-desorption test using the BET method.

Catalyst	A_s (m^2 g^{-1})	Total pore volume (cm^3 g^{-1})
BCFSn-541	0.79	0.0032
BCFSn-631	0.90	0.0035
BCFSn-721	0.80	0.0030
$BaCoO_{3-\delta}$	0.90	0.0032
BCF	1.53	0.0053
BCSn	1.16	0.0038
BCFSn-712	0.84	0.0026
IrO_2	139.8	0.12

Table S3. Oxygen non-stoichiometry (δ) and average valence states of Co and Fe (n) of BCFSn perovskites.

Sample	δ	n
BCFSn-541	3.35(3)	0.29(1)
BCFSn-631	3.32(4)[1]	0.30(4)
BCFSn-721	3.32(0)[2]	0.30(6)

Table S4. Key electrochemical parameters (onset potential, Tafel slope, mass and specific activity at η = 0.45 V) reflecting the OER activity of BCFSn, $BaCoO_{3-\delta}$, BCF, BCSn, BCFSn-712 catalysts as well as the commercial IrO_2.

Catalyst	Onset potential (V vs. RHE)	Tafel slope (mV dec^{-1})	Mass activity @ η = 0.45 V (mA mg$^{-1}_{oxide}$)	Specific activity @ η = 0.45 V (mA cm$^{-2}_{oxide}$)
BCFSn-541	~1.58	76±2	33.7±5.2	4.2±0.7
BCFSn-631	~1.55	68±2	59.4±3.7	6.6±0.4
BCFSn-721	~1.53	69±1	84.5±5.9	10.6±0.7
$BaCoO_{3-\delta}$	~1.55	74±1	17.2±4.0	1.9±0.5
BCF	~1.54	69±2	36.7±2.2	2.4±0.1
BCSn	~1.57	79±1	18.6±5.9	1.6±0.5
BCFSn-712	~1.56	80±2	29.0±9.1	3.5±1.1
IrO_2	~1.47	63±1	130.6±33.4	0.093±0.024

Table S5. Assessment of OER activity for BCFSn catalysts compared with other excellent catalysts reported in the literature.

Catalyst	Activity*		Tafel slope (mV dec^{-1})	Reference
	j @ 1.60 V (mA cm$^{-2}_{oxide}$)	Potential for 1 mA cm$^{-2}_{oxide}$ (V vs. RHE)		
BCFSn	~1.0	~1.58 (for BCFSn-721)	~70	This work
O_2-BSCF	<1.0 (~0.7)	~1.65	~130	[10]
$(Ln_{0.5}Ba_{0.5})CoO_{3-\delta}$	>1.0 (~5)	~1.53 (for $Pr_{0.5}Ba_{0.5}CoO_{3-\delta}$)	~60	[11]
BSCF**	>1.0 (~4)	~1.57	~50	[11]
BSCF**	>10 (~15)	~1.55	~50	[12]

* The activity of BCFSn-721 (either in terms of specific activity at a certain potential, or potential needed to achieve a given current density) is superior to that of O_2-BSCF, but not as good as that of BSCF and $Pr_{0.5}Ba_{0.5}CoO_{3-\delta}$.[10–12] This can be intrinsically correlated with the surface cation (Co species) e_g occupancy of BCFSn-721, lying not very close to the peak of the volcano shape reported by Suntivich et al.[12]

** The differences in the OER activity (*j* @ 1.60 V vs. RHE, or potential for 1 mA cm$^{-2}_{oxide}$) of BSCF reported by the same research group could be attributed to the variation in the specific surface area of BSCF estimated using two different methods (the BET method for the former and the particle size distribution measurements for the latter), which is 0.9 and 0.2 m^2 g^{-1}, respectively. Anyhow, both BSCF oxides exhibited outstanding OER activity.[11,12]

References

[1] Z. Zhang, Y. Chen, M. O. Tadé, Y. Hao, S. Liu, Z. Shao, *J. Mater. Chem. A* **2014**, *2*, 9666.

[2] B. Qian, Y. Chen, M. O. Tadé, Z. Shao, *J. Mater. Chem. A* **2014**, *2*, 15078.

[3] Y. Zhu, C. Su, X. Xu, W. Zhou, R. Ran, Z. Shao, *Chem. Eur. J.* **2014**, *20*, 15533.

[4] A. J. Jacobson, J. L. Hutchison, *J. Solid State Chem.* **1980**, 35, 334.

[5] N. Raghu, V. Ravi, T. R. N. Kutty, *Mater. Res. Bull.* **1991**, *26*, 261.

[6] J.-I. Jung, D. D. Edwards, *J. Solid State Chem.* **2011**, *184*, 2238.

[7] L.-W. Tai, M. M. Nasrallah, H. U. Anderson, D. M. Sparlin, S. R. Sehlin, Solid State Ion. **1995**, *76*, 259.

[8] M. Arnold, Q. Xu, F. D. Tichelaar, A. Feldhoff, *Chem. Mat.* **2009**, *21*, 635.

[9] A. S. Harvey, F. J. Litterst, Z. Yang, J. L. M. Rupp, A. Infortuna, L. J. Gauckler, *Phys. Chem. Chem. Phys.* **2009**, *11*, 3090.

[10] J.-I. Jung, H. Y. Jeong, M. G. Kim, G. Nam, J. Park, J. Cho, *Adv. Mater.* **2015**, *27*, 266.

[11] A. Grimaud, K. J. May, C. E. Carlton, Y. L. Lee, M. Risch, W. T. Hong, J. G. Zhou, Y. Shao-Horn, *Nat. Commun.* **2013**, *4*, 2439.

[12] J. Suntivich, K. J. May, H. A. Gasteiger, J. B. Goodenough, Y. Shao-Horn, *Science* **2011**, *334*, 1383.
